# Study of Edible Plants: Effects of Boiling on Nutritional, Antioxidant, and Physicochemical Properties

**DOI:** 10.3390/foods9050599

**Published:** 2020-05-08

**Authors:** José Arias-Rico, Francisco Jesús Macías-León, Ernesto Alanís-García, Nelly del Socorro Cruz-Cansino, Osmar Antonio Jaramillo-Morales, Rosario Barrera-Gálvez, Esther Ramírez-Moreno

**Affiliations:** 1 Área Académica de Enfermería, Instituto de Ciencias de la Salud, Universidad Autónoma del Estado de Hidalgo, Pachuca 42160, Hidalgo, Mexico; josearias.rico@hotmail.com (J.A.-R.); osmar_jaramillo@uaeh.edu.mx (O.A.J.-M.); rosariobag@hotmail.com (R.B.-G.); 2 Área Académica de Nutrición, Instituto de Ciencias de la Salud, Universidad Autónoma del Estado de Hidalgo, Pachuca 42160, Hidalgo, Mexico; jesuispaco@hotmail.com (F.J.M.-L.); ernesto_alanis@uaeh.edu.mx (E.A.-G.); ncruz@uaeh.edu.mx (N.d.S.C.-C.)

**Keywords:** Mexican plants, boiling, nutritional composition, physicochemical properties

## Abstract

The consumption of vegetables in Mexico includes a wide variety of plants that grow naturally as weeds in the fields. The intake of these vegetables is very important in the Mexican diet because these plants supply an important input of nutrients and compounds such as fiber, vitamins, minerals, and antioxidants. Thus, the plants may be universally promoted as healthy. However, there is little information about these vegetables of popular consumption, especially in terms of the nutritional changes caused by boiling. To determine the influence of boiling on five plants of popular consumption in Mexico, the nutritional composition (proximal analysis, dietary fiber, and oxalates), antioxidant compounds (ascorbic acid, phenolics), antioxidant activity (measured by ABTS and DPPH assays), and physicochemical characteristics (water retention capacity, viscosity, color, and SEM) were evaluated. The boiling affected the nutritional composition of plants, mainly soluble compounds as carbohydrates (sugars and soluble fiber), ash, ascorbic acid, and phenolic compounds and caused changes in food hydration and color. Therefore, it is recommended that these plants be consumed raw or with short boiling times and included the cooking water in other preparations to take advantage of the nutrients released in the food matrix. In the future, to complete studies, 3 to 5 min of cooking should be considered to minimize undesirable modifications in terms of the vegetables’ composition.

## 1. Introduction

The traditional Mexican diet may be of great interest because it includes the highest number of endemic plants. Between 20% and 30% of plant species in Mexico contribute to the basic diet of low-income populations [1,2]. *Chenopodium* spp., *Suaeda torreyana*, *Portulaca oleracea*, and *Porophyllum* spp. are some of the plants commercially available and of high consumption in Mexican gastronomy in raw and cooked preparations [1]. The autochthonous plants are accessible, little known, easy to cook, and have a high content of nutrients (vitamins, minerals, fiber) and bioactive compounds. The plants could provide an alternative ingredient as compared to other foods due to their vegetal origin designed for the therapy of severe acute malnutrition or other diseases [3].

More of the leafy vegetables are consumed after a process that involves the re-motion of the edible parts, washing, cutting, and cooking in boiled water. It is known that cooking could induce positive or negative changes related to the loss of nutritional or bioactive components in the cooking water, softening of the tissue, color change, aroma formation, and inactivation of compounds considered as anti-nutritional factors improving the bioavailability of these components [4]. These changes are related to the breakdown of food matrix (mainly formed of dietary fiber). Initially, the release of compounds with low molecular weight and water, which could be trapped or released of this food matrix [5]. The compounds released during heat treatment could form complexes between fiber or other nutrients of the food [6], be exposed to reactions of degradation (oxidation of vitamin C), have a higher bioavailability of some nutrients released by their transformation into active structures [5,7], or could decrease compounds considered anti-nutrients (oxalic acid, phytic acid, etc.). These modifications will influence the nutritional value and sensorial properties of cooked vegetables depending upon the differences of process conditions as well as nutritional and morphological characteristics of vegetables [8].

Although the information available regarding some of these plants is mainly about their relationship with traditional medicine or food composition tables, much of its nutritional composition or compounds considered as anti-nutrients after of heat treatment is unknown. Therefore, it is important to enhance the consumption of plants that belong to the Mexican culture, which could improve the health status of this population [9]. This study aimed to assess the effect of cooking on the nutritional, antioxidant characteristics and structural changes of five edible plants from México.

## 2. Materials and Methods

### 2.1. Plant Materials

The plants studied were *Chenopodium Nuttalliae* Safford (Huazontle), *Suaeda Torreyana* S. Watson (Romerito), *Portulaca Oleracea* L. (Verdolaga), *Chenopodium Album* L. (Quelite), and *Porophyllum Ruderale* (Jacq.) Cassini (Asteraceae) (Papaloquelite). A single batch of these plants were purchased from central supermarket in Pachuca, Hidalgo Mexico. Although the production of these plants is carried out year-round, the samples were obtained in September. In general, these plants could be recollected from producers in the same population or its surroundings. The descriptions of these plants are presented in Table 1.

#### Sample Preparation

**Figure 1:**
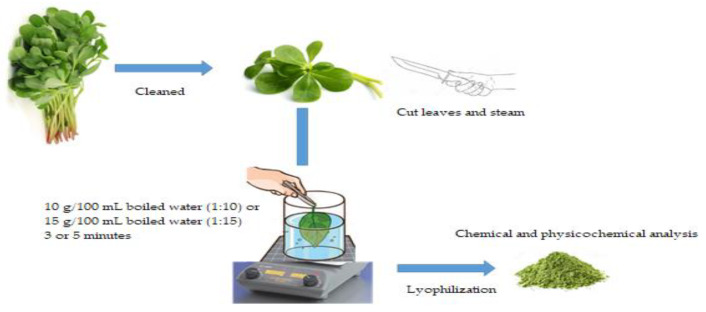
Preparation procedure of plants.

All plants were cleaned and non-edible portions were discarded. An edible part of these plants (leaves and stems) was maintained in raw and another part was boiled with the exception of *Porophyllum Ruderale* (Jacq.) Cassini (Asteraceae), which was evaluated as raw. Boiling conditions were performed by preliminary experiments carried out for each vegetable, considering the minimum cooking time to reach a similar softness, palatability, and taste according to the Mexican consumption habits (Table 2). Each plant batch was divided into three parts to have at least three repetitions in the experiments. A total of 10 g of plant was chopped and boiled in a beaker with 100 or 150 mL of distilled water (1:10 or 1:15 food/water) (Figure 1) to complete the cooking. The boiling water was drained off for 60 s. Raw and cooked plants were freeze-dried, ground to 500 mm mesh, and stored at −20 °C for posterior analysis in black bags. All determinations of proximal analysis, antioxidant, and physicochemical properties were performed in lyophilized samples at least in triplicate. Results of processed samples have been corrected considering a factor that takes into account the soluble solids’ loss due to changes of moisture after processing [11].

### 2.2. Nutritional Characteristics

#### 2.2.1. Proximal Analysis

The proximal analysis of lyophilized samples was determined using AOAC (Association of Analytical Communities, 2005) methods [12]. The moisture was estimated by a weight difference after drying (method 925.09). The protein content was estimated by the Kjeldahl procedure (method 950.48). The fat was extracted with petroleum ether from dry samples using a Soxhlet extraction system (Büchi Labortechnik AG, Flawil, Switzerland) (method 983.23) and ash content was determined after incineration of the sample at 550 °C ± 10 °C (method 930.05). Lastly, carbohydrates were calculated by difference of the proximate parameters and total dietary fiber, as shown in the next formula. All the results were expressed as grams of each compound per 100 g of dried basis (g/100 g db).

Soluble carbohydrates=100−protein−lipids−ash−TDF

#### 2.2.2. Total Dietary Fiber (TDF)

Soluble (SDF) and insoluble (IDF) dietary fibers were determined according to AOAC [12], with an enzymatic–gravimetric method using a Total Dietary Fiber Assay Kit (Sigma TDF-100A Kit, Sigma). The sum of soluble dietary fiber (SDF) and insoluble dietary fiber (IDF) was considered as total dietary fiber (TDF).

#### 2.2.3. Total Oxalate

The analysis of oxalic acid was performed by High Performance Liquid Chromatography (HPLC) [13]. An amount (500 mg) of lyophilized sample was extracted using 25 mL of metaphosphoric acid solution (45 g/L) to determine total oxalate or 25 mL of distilled water to soluble oxalate with magnetic stirring for 15 min. The sample was centrifuged (30 min, 8960 g) and the supernatant was collected and aforated to 25 mL with metaphosphoric acid or distilled water. Extracts were chromatographed on a Sphereclone ODS-column with 1.8 μmol/L H_2_SO_4_ in distilled water (pH 2.6) as eluent in a Waters HPLC system equipped with a UV-visible detector. Aliquots of extracted samples (20 μL) were injected at a flow rate of 0.4 mL/min. The retention time considered for oxalic acid was 4.5 min and the area of the chromatographic peak was monitored at 215 nm. The standard of oxalic acid dehydrate (Sigma) was used to determine oxalates. The insoluble oxalate content was calculated as the difference of total oxalate (metaphosphoric acid extract) with soluble oxalate (distilled water extract).

### 2.3. Antioxidant Compounds

#### 2.3.1. Extraction of Antioxidants

The extraction of dried plants was carried out following an efficient extraction of antioxidants with two extraction cycles performed with aqueous-organic solvents of different polarities [14,15]. It was used to determine ascorbic acid, total phenolic content, and antioxidant capacity. The methodology consists in the extraction of 0.25 g of the sample with 10 mL of methanol/water (50:50, *v*/*v*) and stirred for 1 h and centrifuged (6678× *g*, 10 min) (VanGuard V6500 Hamilton Bell^Ⓡ^, Montvale, NJ, USA). The supernatant was transferred to a flask of 25 mL. The residue was re-extracted with 10 mL of acetone:water (70:30, *v*/*v*) and centrifuged again. Then, the combination of both supernatants was carried out and the flask was aforated to 25 mL using previously prepared solutions of methanol and acetone (50:50, *v*/*v*).

#### 2.3.2. Ascorbic Acid (AA)

The ascorbic acid content was determined following the colorimetric method described by N. Dürüst [16]. In brief, 100 μL of sample was mixed with 100 μL of acetate buffer and 800 μL of DCPI (2,6-dichlorophenol indophenol). After the reaction mixture was conducted, the absorbance was measured in a microplate reader (Power Wave XS UV-Biotek, software KC Junior, Kansas, MO, USA) at 520 nm using blank oxalic acid with a concentration of 0.4%. A standard curve of ascorbic acid was obtained with concentrations of 0–50 mg/L of ascorbic acid to determine the concentration of the sample as milligrams of ascorbic acid per 100 g on a dried basis (mg AA/100 g db).

#### 2.3.3. Total Phenolic Compounds (TPC)

The amount of total phenolic compounds was assessed according to the Folin-Ciocalteau methodology [17]. The reaction mixture was comprised of 100 µL of antioxidant extract and the same amount of Folin–Ciocalteau reagent. After 3 min at room temperature, 2 mL of sodium carbonate solution (75 g/L) and 2.8 mL of water was added and mixed. The absorbance of the solution was measured (750 nm) after an hour of the reaction using a microplate reader. A linear curve was obtained with concentrations of 0–300 mg/L of gallic acid used as a reference standard. The results were expressed as milligrams of gallic acid equivalents per 100 g on a dried basis (mg GAE/100 g db).

### 2.4. Antioxidant Capacity

#### 2.4.1. ABTS

The antioxidant capacity measured by ABTS was determined according to the Kukoski [18] methodology. A stock solution of the radical ABTS·+ was produced with the reaction mixture of 7 mmol/L ABTS (2,2′-azino-bis-(3-ethylbenzothiazoline-6-sulphonic acid diammonium salt) and 2.45 mmol/L of potassium persulfate under room temperature and dark conditions. This solution was kept in dark conditions at room temperature and it stood for 16 h. The ABTS solution was diluted with deionized water until getting an absorbance of 0.70 ± 0.10 at 754 nm. An aliquot of 20 µL of the plant extract was added to 980 µL of diluted ABTS radical solution and kept at room temperature for 7 min. The final absorbance was measured at 754 nm in a microplate reader (Power Wave XS UV-Biotek, software KC Junior, Kansas, MO, USA). Solutions of a known concentration of Trolox (6-hydroxy-2,5,7,8-tetramethylchroman-2-carboxylic acid) (0–50 μmol/L) were used to determine the antioxidant capacity and it was expressed as micromole of Trolox equivalents per 100 g on a dried basis (µmol TE/100 g db).

#### 2.4.2. DPPH

The ability of antioxidants to scavenge free radicals was measured according to Morales and Jimenez-Perez [19] using DPPH (1,1-diphenyl-2-picrylhydrazyl). A stable DPPH radical was prepared using an ethanolic solution with a concentration of 7.4 mg/100 mL. After that, 100 µL of antioxidant extract were mixed with 500 µL of DPPH solution and the mixture was kept at room temperature for 1 h. The solution was shaken and centrifuged (3000 rpm, 10 min) to measure the absorbance using a microplate reader at 520 nm. Antiradical activity was measured with a linear standard curve of Trolox (3.75 mg/50 mL of ethanol) with concentrations of 0–300 µmol/L and expressed as micromole of Trolox equivalents per 100 g on a dried basis (µmol TE/100 g db).

### 2.5. Physicochemical Characteristics

#### 2.5.1. Water Retention Capacity (WRC) and Viscosity

The water retention capacity was measured according to Robertson et al. [20]. To measure the viscosity, a dispersion with distilled water (3%) was obtained from each lyophilized sample using a Brookfield DV-E viscometer at temperatures of 27 °C.

#### 2.5.2. Color

The color parameter was carried out in a Hunter Lab system with a colorimeter (Minolta CR300 Japan) on the basis of L*, a*, and b* values. L* parameter indicates lightness (lightness, black = 0, white = 100), a* the degree of red-green color (redness > 0, greenness < 0), and b* values (yellowness > 0, blue < 0) degree of the yellow-blue color [21].

#### 2.5.3. SEM (Scanning Electron Microscopy)

To determine the microstructure of plants, scanning electron microscopy (SEM) with microanalysis was used by energy dispersion of X-ray (JSM 6300, Jeol, Japan), which was equipped with an X-Ray spectrometer (Noran) by energy dispersion (EDS) (WD = 15 mm, accelerating voltage = 15 kV and acquisition time 100 s). The amount of dried sample was placed on specimen holders with a “Leit-C” cover with gold by sputtering and observed in a STEREO SCAN Mk 1.

### 2.6. Statistical Analysis

All the determinations were carried out in triplicate and the results of the evaluated samples (*n* = 3) were expressed by mean ± standard error of the mean (SE). The differences between the samples were analyzed by performing an one-way analysis of variance (ANOVA) using a Tukey test (*p* < 0.05). To determine the difference and the levels of statistical significance between raw and cooked vegetables, a student *t*-test was used. Both analyses were performed in an SPSSSystem (WINTM version 15.0).

## 3. Results and Discussion

### 3.1. Nutritional Composition

In the study of cooked vegetables, the diffusion of water and low molecular compounds of the food matrix could affect the nutritional composition of the food. On the other hand, the released compounds could have degradation reactions or may transform to bioavailable structures [4,5,6]. In this study, the edible parts of evaluated plants were composed of a high content of moisture (85%–93%), which is characteristic of vegetable products (Table 3). The dry matter was characterized mainly by carbohydrates (19%–52%), protein (26%–31%), dietary fiber (19%–26%), a low content of lipids (5%–22%), and ashes (0.2%–0.7%). Raw *Portulaca Oleracea* L. was characterized by high content of moisture, proteins, lipids, and total dietary fiber, while *Chenopodium album* L. was characterized by a high content of proteins, ash, and dietary fiber. *Porophyllum ruderale* (Jacq.) Cassini presented a high level of carbohydrates and ash. These data were according to the results reported in other studies with vegetables [22]. The lipids data showed the higher variability, which had the highest value *Portulaca oleracea* L. and the lowest *Porophyllum ruderale* (Jacq.) Cassini (Asteraceae).

All the plants were evaluated after thermal treatment, except the *Porophyllum ruderale* (Jacq.) Cassini (Asteraceae) plant, which is consumed raw as a condiment in different dishes. In all the plants, the boiling caused a loss of nutrients (around 2% to 16%), while *Suaeda torreyana* S. Watson was the plant with the most important loss of nutrients (around 10%–38%). Lipids and proteins were strongly affected in *Chenopodium album* L. and *Portulaca oleracea* L. while the dietary fiber in *Chenopodium nuttalliae Safford* and *Suaeda torreyana* S. Watson was affected. These losses could be due to the hydrolysis and release of compounds of low molecular weight as carbohydrates and ashes into the cooking water [23].

Raw plants were characterized by a high content of TDF, where IDF was the most important fraction (Table 4). The TDF was modified by the thermal processing, which decreased SDF while the IDF fraction remained in almost all samples. According to Nyman [24], during thermal processing of vegetables, some glycosidic linkages may be broken and the dietary fiber polysaccharides of the cell wall structure may be depolymerized by forming protein-fiber complexes [25] or affecting the solubility [26]. This last could cause the decrease or increase of these compounds. However, a significant increase of TDF content in plants compared to their raw forms may be beneficial, as insoluble fiber has been shown to be closely related to glycemic responses in the body [27].

### 3.2. Oxalic Acid

The function of oxalate in plants is involved in seed germination, ion balance, calcium and magnesium storage, detoxification, insect repulsion, and structural resistance [28]. Therefore, more than 90% of the total calcium of a plant can be found as oxalate salt [29,30]. The main food sources of this component are spinach and rhubarb [28]. The spinach is considered a vegetable with a high content of oxalic acid with values between 400–900 mg/100 g fw (fresh weight). In comparison with the spinach, the studied plants had a medium content of oxalates (around 94 to 161 mg/100 g fw or 831 to 1524 mg/100 g db) while the *Porophyllum ruderale* (Jacq.) Cassini (Asteraceae) plant had the lower content of total oxalates (15.80 mg/100 g fw or 107.58 mg/100 g db) (Table 5).

Most of studied samples had a significative loss of total oxalates (17%–48%) characterized by the loss of soluble oxalates into cooking water (around 31% to 48%), while *Suaeda torreyana* S. Watson had the most important loss of these compounds (76%). On the other hand, insoluble oxalates remained in a higher amount in the food matrix (losses of 6% to 35% only), or even had an increase of this component as in *Portulaca oleracea* L. These findings were consistent with different studies [31,32], which found that boiling reduced the total oxalate of vegetables by leaching a high amount of soluble oxalate into the cooking water, while the insoluble oxalate remained in the food matrix or had moderate losses. These last studies had reported an increase (between 25% and 40%) of insoluble oxalate after boiling of vegetables as potatoes [32], silver beet steams (*Beta vulgaricus v. cicla*), and parsnip (*Peucadenum sativum*) [31]. The decreases of total oxalate after boiling in this study is significant because total oxalate in vegetable foods is inversely correlated with the calcium bioavailability [31,33].

### 3.3. Antioxidant Properties

#### 3.3.1. Ascorbic Acid

Ascorbic acid is a constituent of all vegetable foods and edible plants, and it is easy to achieve the requirement of this vitamin in the daily diet (60 mg per day for adults). Other studies had determined that more than 400 mg/day can improve protection against oxidative stress, certain cancers, degenerative diseases, and non-communicable diseases [34].

The studied plants had an ascorbic acid content (273.1 to 810.6 mg AA/100 g db or 37.4–48.6 mg AA/100 g fw) (Table 6), similar to other plants as cabbage, spinach, watercress, cauliflower, and kale [34]. Only the *Porophyllum ruderale* (Jacq.) Cassini (Asteraceae) plant had the higher content of this component (952.2 mg/100 g db or 140 mg/100 g fw), which is similar to broccoli or Brussel sprouts [35]. Boilining among all the plants reduced ascorbic acid (by around 35%). *Portulaca oleracea* L. was the most strongly affected (62%). During heat treatment, ascorbic acid could be affected by the high solubility, heat sensibility, and oxidation process in fruit and vegetables [8]. In addition to the significant loss of this component during storage, preparation, and cooking, the availability of fruits and vegetables considered as sources of ascorbic acid are factors that should be considered, mainly in populations where the diets are lacking this nutrient [35].

#### 3.3.2. Total Phenolic Compounds (TPC)

According to Nicoli et al. [8], the TPC content may increase, decrease, or remain unchanged after thermal processing of fruits or vegetables. The polyphenol content of the plant studied ranged from 960.8 to 1477.2 mg GAE/100 g db (Table 6). Values of total phenolic were similar to vegetables (430–1285 mg/100 g db) [9,36] or within leafy plants (80 to 6722 mg gallic acid/100 g db) [37,38,39]. Few studies had characterized the phenolic compounds of the leafy vegetable. Lagari et al. [40] found vainillinic, caffeic, syringic acids and vanillin in the leaves of *Chenopodium album* L. This information of phenolic compounds is more complete and allows a comparison of more objective between treatments. The Folin-Cioucalteau method could have possible interferences with amino acids or with solvents and could provide a false positive [41]. Hence, it shows the importance of studying these samples with finer methodologies as chromatography or mass spectrometry, among others.

The heat treatment caused a slight decrease on the phenolic content of studied samples (losses ≈ 11% to 15%), and only *Suaeda torreyana* S. Watson plant had the most important loss (30%). According to other studies, food processing causes the disruption from cellular structures such as lignin and polysaccharides and release of phenolic compounds in cooking water [42].

#### 3.3.3. Antioxidant Capacity

To evaluate the effectiveness of antioxidants in samples, two methods (ABTS and DPPH) were chosen (Table 6). The antioxidant activity measured by ABTS had values between 192.1 and 437 µmol TE/100 g db, while DPPH was ≈2300 µmol TE/100 g db. The *Porophyllum ruderale* (Jacq.) Cassini (Asteraceae) plant had the highest values of antioxidant capacity (437 µmol TE/100 g db for ABTS and 6355 µmol TE/100 g db for DPPH) related to a high content of ascorbic acid and phenolic compounds, which is relevant since this plant is consumed raw.

In this study, we found a diminution of ascorbic acid and phenolic compounds by boiling, according to other studies of conventional vegetables [42,43,44]. Nicoli et al. [8], had established that the processed foods could decrease the overall antioxidant potential, as a consequence of the loss of natural antioxidants or formation of novel compounds with pro-oxidant activity. Therefore, the decrease of antioxidant capacity of the studied samples by boiling ranged between 6% to 65% (Table 6). The samples most affected were *Portulaca Oleracea* L. (decrease of 62%), possibly by the diminution of ascorbic acid and *Suaeda Torreyana* (reduction of 65%) related to the loss of phenolic compounds.

### 3.4. Physicochemical Properties

#### 3.4.1. Water Retention Capacity (WRC) and Viscosity

During the cooking process, the cell wall performed changes mainly in polysaccharides as dietary fiber affected the physicochemical properties of the sample. Most of these changes also have nutritional effects. The studied plants had a high content of insoluble fiber, and, due to this, they are characterized by cellulose. Residual polysaccharides can adsorb water and hydrate the fibrous matrix, without forming viscous solutions as soluble fibers [45]. This hydrated matrix of insoluble fiber has many physiological benefits such as increasing fecal bulk with the consequent reduction of gastrointestinal transit time [46]. The hydration property of a sample could be measured as WRC of the dietary fiber. The values of WRC (8.4 to 12.4 g water/g db) were similar to apple fiber with values of 3.8 to 7.1 g/g db [45]. However, these values are low compared to apple pectin (16.51 g/g db) [47]. The values of WRC of the samples were not affected by thermic treatment. However, the values of viscosity (2.56–7.68 MPa) in the samples evaluated did decrease after boiling around 21% to 51%. According to Marcotte et al. [48], the loss of viscosity suggests an irreversible structural breakdown of the gel affected by the disruption of the cell structure.

#### 3.4.2. Color

The fresh appearance and green color of vegetable foods are key characteristics for a better acceptation of the product between the consumers. The results of color parameters are shown in Table 7. The studied plants were in quadrant green (−a) and yellow (+b) and values of luminosity (L*) between 24.1 to 34.9. *Chenopodium nuttalliae* Safford had the highest values in luminosity (34.9 ± 0.8) and b* (22.9 ± 0.1) parameter. It can be seen that cooking the plants reduced the color parameters significant to *Portulaca oleracea* L. and *Chenopodium nuttalliae* Safford. The results above indicate that raw samples have a light green color and heat treatment caused a darker color due to the conversion of the chlorophyll structure to pheophytin, which provides colors as a green olive [49,50].

#### 3.4.3. SEM (Scan Electronic Micrography)

Foods safety and sensorial characteristics require thermal treatment of raw food during food processing on an industrial food and home level. However, these heat treatments could affect the cell structure and, therefore, the nutritional composition.

**Figure 2:**
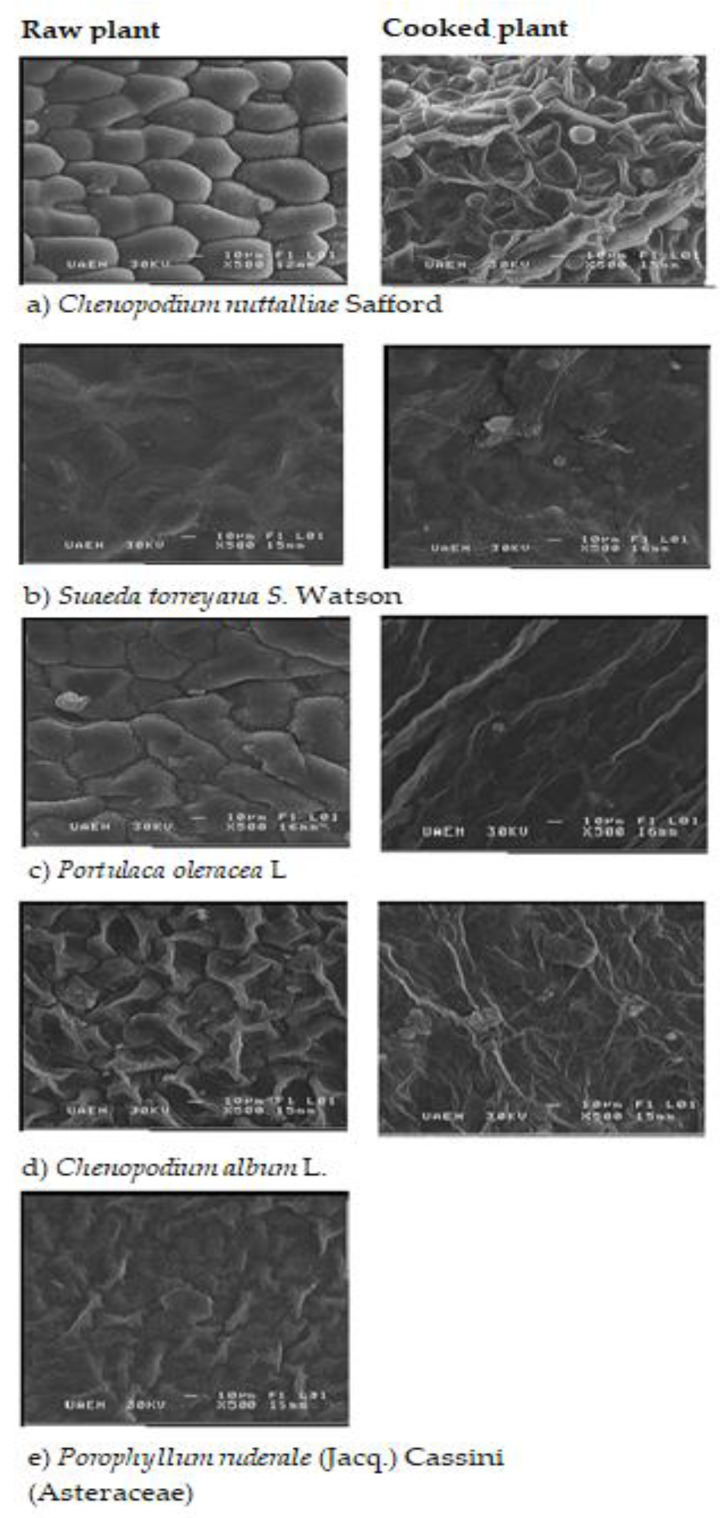
Scanning electron microscopy (SEM) of raw (left photography) and boiling (right photography) tissue of edible plants.

Figure 2 shows the micrography of the plants’ structure studied. In the right part, the tissue of raw plants is observed, which is characteristic of irregular polygonal and parenchymatous cells. The plants *Chenopodium nuttalliae* Safford and *Portulaca oleracea* L. showed intercellular spaces, according to Grote and Fromme [51], while the other studied plants formed a network. After heat treatment, this organized structure was lost due to breakage of the cell wall. The cells appear to be more tightly packed and there was more amorphous extracellular material. These structural changes are caused by the effects in the cell-wall polysaccharides (esterification grade, loss of pectin, and release of soluble sugars), non-starch polysaccharides, and proteins, which could be related to the considerable amount of solid matter of plants lost in the boiling water [52,53,54] affecting the physicochemical properties of dietary fiber, which causes deleterious nutritional effects [8]. Therefore, it is important to consider that dishes or food preparation include heat treatments of vegetables carried out during long periods of time. Thus, it is important to consider short periods between 3 and 5 min to take advantage of their nutritional properties without losing its sensory acceptance.

## 4. Conclusions

The edible plants studied had an appreciable content of dietary fiber, ashes, ascorbic acid, phenolic compounds, and a good antioxidant capacity. However, boiling for 3 to 5 min caused the loss of soluble compounds (sugars, soluble fiber, ash, ascorbic acid, and phenolic compounds), and affected the color, hydration properties, and cell integrity. Hence, it is important to recommend the use of boiling water of vegetables in other food preparations to take advantage of the released soluble compounds. Due to the study of a food could be overvalued when only data of raw product are studied, it is important to consider short periods of heat treatment of leafy vegetables in future studies to reduce the nutrient losses without affecting its sensory acceptance.

## Figures and Tables

**Table 1:** Botanical characteristics of studied plants [10].

Plants	*Scientific Name* (Common Name)
* 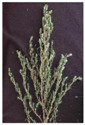 *	*Chenopodium Nuttalliae* Safford (Huauzontle, quelite cenizo) Herbaceous plant of erect bearing with a prominent, angular stem and with grooves. The stem presents branching and its branches emerge obliquely from the main stem. The number of primary branches ranges from 32 to 41. The leaves have a serrated edge generally of 3 to 12 teeth. The inflorescences are amarantiform. This plant is considered to be a condiment or the main ingredient in Mexican regional food, which is available throughout the year.
* 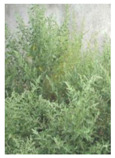 *	*Suaeda torreyana* S. Watson (Romerito) The perennial shrub can reach a height of two meters. Its stems are woody and erect with many branches and firm needles. Its color is dark green in the beam and white at the bottom. The flowers vary in their strain and their small seeds have a camphoric aroma. It is an aromatic plant used in food preparation and is developed in the base of the Mexican mountains, where humidity is the main factor for development of arboreal species throughout the year but mainly from May to September.
* 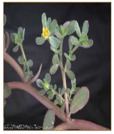 *	*Portulaca oleracea* L. (Verdolaga) It has a smooth stem and red branches. Alternate leaves, obovate-cuneate to spatulated, 0.5–3 cm long, and 0.2–1.5 cm wide. Sessile flowers, solitary or grouped by few, are surrounded by scarce (sometimes none) inconspicuous hairs. Oval to orbicular sepals are 2.5–4.5 mm long and wide and somewhat caked. The yellow petals are 3–5 mm long. There are 6 to 10 stamens in which 4 to 6 are lobed. The seeds are formed in a small pod, which opens when the seeds are ripe. The consumption of this plant is in an unmatured state and could be found in irrigation crops throughout the year, while, in seasonal crops, the production is from March to August.
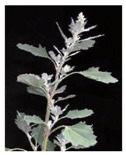	*Chenopodium Album* L. (Quelite) This plant is similar to grass up to 70 cm tall, erect, and red-colored. It has elongated and pointed leaves. Its flowers are greenish, small, and are grouped in long spikes. This plant contributes with different aromas, colors, and flavors to the human diet and it is used in many cases as an ingredient of dishes or a condiment available throughout the year.
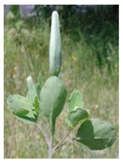	*Porophyllum ruderale* (Jacq.) Cassini (Asteraceae) (Papaloquelite) It is a straight plant that generally grows between 20 and 100 cm in height. The leaves are a little divided and pale green and the flowers are yellow. This specie is an herb with a strong and characteristic flavor. The leaves and stems are commonly consumed raw. It is used as salad dressings as a condiment in some rural and semi-rural regions of Mexico. It is available all year but is mainly found from June to October.

**Table 2:** Boiling conditions of the studied plants.

Scientific Name Plant	Common Name	Edible Parts	Time (min)	Dilution
*Chenopodium nuttalliae* Safford	Huazontle	Leaves and stems	5	1:15
*Suaeda torreyana* S. Watson	Romerito	Leaves and stems	3	1:10
*Portulaca oleracea* L.	Verdolaga	Leaves and stems	5	1:10
*Chenopodium album* L.	Quelite	Leaves and stems	5	1:15
*Porophyllum ruderale* (Jacq.) Cassini (Asteraceae)	Papaloquelite	Leaves and stems	Raw	Raw

**Table 3:** Effect of boiling on proximal analysis of edible plants (g/100 g db).

*Scientific Name of Plant*	Treatment	Moisture	Proteins	Lipids	Ash	Total Dietary Fiber	Carbohydrates ^B^
*Chenopodium nuttalliae* Safford	Raw	86.3 ± 0.4 ^a^	30.1 ± 0.0 ^f^	10.1 ± 1.3 ^b^	0.6 ± 0.1 ^abcd^	22.4 ± 0.3 ^c*^	36.9 ± 1.3 ^d^
	Boiled ^A^	85.6 ± 0.2 ^a^	29.4 ± 0.0 ^e^	9.4 ± 0.7 ^b^	0.5 ± 0.0 ^abcd^	18.8 ± 1.2 ^a^	42.0 ± 0.5 ^e^
*Suaeda torreyana* S. Watson	Raw	88.8 ± 0.6 ^b*^	31.3 ± 0.4 ^h*^	19.2 ± 0.8 ^ef^	0.6 ± 0.3 ^abcd*^	24.9 ± 0.3 ^d*^	24.0 ± 1.2 ^b*^
	Boiled ^A^	91.8 ± 0.0 ^c^	23.6 ± 0.1 ^a^	17.1 ± 0.3 ^cd^	0.4± 0.1 ^abc^	18.5 ± 0.6 ^a^	40.4 ± 0.2 ^e^
*Portulaca oleracea* L.	Raw	94.0 ± 0.3 ^d^	30.7 ± 0.1 ^g*^	22.8 ± 0.7 ^g*^	0.3 ± 0.0 ^ab^	26.9 ± 1.6 ^e^	19.3 ± 0.7 ^a^
	Boiled ^A^	90.7 ± 0.1 ^c^	26.1 ± 0.2 ^b^	20.7 ± 0.5 ^f^	0.2 ± 0.8 ^a^	29.2 ± 0.0 ^f^	23.8 ± 0.3 ^b^
*Chenopodium album* L.	Raw	88.4 ± 0.2 ^b*^	30.8 ± 0.1 ^gh^	18.6 ± 0.2 ^de*^	0.8 ± 0.0 ^d^	26.3 ± 1.7 ^e^	23.7 ± 1.0 ^b^
	Boiled ^A^	90.5 ± 0.1 ^c^	27.8 ± 0.0 ^d^	16.2 ± 0.7 ^c^	0.7 ± 0.1 ^cde^	25.3 ± 0.9 ^d^	30.3 ± 0.4 ^c^
*Porophyllum ruderale* (Jacq.) Cassini (Asteraceae)	Raw	85.3 ± 0.8 ^a^	26.9 ± 0.0 ^c^	5.0 ± 0.3 ^a^	0.6 ± 0.2 ^cd^	19.7 ± 0.6 ^b^	47.4 ± 0.3 ^f^

Values are mean ± SE (*n* = 3). ^a–h^ Different letter in the same column indicate a significant difference (*p* ≤ 0.05) between the samples. ^A^ Corrected value taking into account the soluble solid loss during cooking. * The asterisk indicates a significant difference between the raw sample and the boiled sample. ^B^ Carbohydrates were calculated (100 -protein, ash, lipids, and total dietary fiber (TDF)).

**Table 4:** Effect of boiling on soluble and insoluble fiber of edible plants (g/100 g db).

*Scientific Name Plant*	Treatment	Dietary Fiber Fractions	
		Soluble	Insoluble
*Chenopodium nuttalliae* Safford	Raw	10.6 ± 0.5 ^e*^	11.7 ± 0.4 ^a^
	Boiled ^A^	6.1 ± 1.0 ^d^	12.6 ± 1.2 ^a^
*Suaeda torreyana S.* Watson	Raw	0.4 ± 0.0 ^a^	24.6 ± 0.3 ^d*^
	Boiled ^A^	0.2 ±0.0 ^a^	18.3 ± 0.6 ^c^
*Portulaca oleracea* L	Raw	4.7 ±0.3 ^c^	22.3 ± 1.6 ^c^
	Boiled ^A^	4.3 ± 0.4 ^c^	24.9 ± 0.0 ^d^
*Chenopodium album L.*	Raw	1.0 ± 0.1 ^b^	25.3 ± 1.7 ^d*^
	Boiled ^A^	0.7 ± 0.1 ^b^	24.6 ± 1.0 ^d^
*Porophyllum ruderale* (Jacq.) Cassini (Asteraceae)	Raw	4.9 ± 0.6 ^c^	14.9 ± 0.6 ^b^

Values are mean ± SE (n = 3). ^a–d^ Different letter in the same column indicate a significant difference (*p* ≤ 0.05) between the samples. ^A^ Corrected value taking into account the soluble solid loss during cooking. * The asterisk indicates a significant difference between the raw sample and the boiled sample.

**Table 5:** Effect of boiling on soluble and insoluble oxalate of edible plants (mg/100 g db).

*Scientific Name Plant*	Treatment	Soluble	Insoluble
*Chenopodium nuttalliae* Safford	Raw	985.2 ± 27.6 ^f*^	197.6 ± 58.3 ^b*^
	Boiled^A^	678.7 ± 78.1 ^e^	Traces
*Suaeda torreyana* S. Watson	Raw	598.3 ± 35.0 ^d*^	242.5 ± 52.3 ^c*^
	Boiled^A^	137.1 ±2.9 ^b^	155.7 ± 4.9 ^a^
*Portulaca oleracea* L.	Raw	1045.8 ±3.7 ^g*^	478.3 ± 16.3 ^e*^
	Boiled^A^	538.2 ± 11.1 ^d^	749.9 ± 10.9 ^f^
*Chenopodium album* L.	Raw	623.9 ± 20.4 ^e*^	324.5 ± 16.1 ^d*^
	Boiled^A^	326.2 ± 4.4 ^c^	303.7 ± 11.9 ^d^
*Porophyllum ruderale* (Jacq.) Cassini (Asteraceae)	Raw	107.6 ± 1.8 ^a^	Traces

Values are mean ± SE (*n* = 3). ^a–g^ Different letter in the same column indicate a significant difference (*p* ≤ 0.05) between the samples. ^A^ Corrected value taking into account the soluble solid loss during cooking. * The asterisk indicates a significant difference between a raw and a boiled sample.

**Table 6:** Effect of boiling on ascorbic acid, total phenolic compounds, and antioxidant activity (ABTS and DPPH) of edible plants.

*Scientific Name Plant*	Treatment	Ascorbic Acid (mg AA/100 g db)	Total Phenolics (mg GAE/100 g db)	ABTS (µmol TE/100 g db)	DPPH (µmol TE/100 g db)
*Chenopodium nuttalliae* Safford	Raw	273.1 ± 5.9 ^ab*^	1463.3 ± 9.6 ^f*^	326.7 ± 3.4 ^d^	2440.7 ± 245.0 ^e*^
	Boiled ^A^	172.2 ± 4.9 ^a^	1268.4 ± 12.9 ^d^	304.6 ± 3.4 ^d^	1864.8 ± 172.0 ^d^
*Suaeda torreyana* S. Watson	Raw	434.2 ± 14.4 ^b*^	1081.4 ± 25.4 ^b*^	325.9± 2.7 ^d*^	1957.4 ± 198.5 ^e^
	Boiled ^A^	293.0 ± 5.8 ^ab^	753.0 ± 15.5 ^a^	122.3± 2.4 ^a^	1591.2 ± 285.7 ^b^
*Portulaca oleracea* L.	Raw	810.6 ± 11.8 ^c*^	1477.2 ± 83.3 ^g*^	250.5 ±3. 4^c*^	2526.4 ± 337.0 ^f*^
	Boiled ^A^	305.9 ± 4.6 ^ab^	1310.7 ± 12.3 ^e^	170.2 ± 4.6 ^ab^	875.3 ± 95.6 ^a^
*Chenopodium album* L.	Raw	420.3 ± 8.3 ^b*^	1123.1 ± 17.3 ^c*^	192.1 ± 2.7^b^	2378.2 ± 152.3 ^e*^
	Boiled ^A^	276.0 ± 11.1 ^b^	950.5 ± 8.4 ^a^	148.8 ±15.7^ab^	1694.9 ± 28.1 ^c^
*Porophyllum ruderale* (Jacq.) Cassini (Asteraceae)	Raw	952.2 ± 16.7 ^c^	960.8 ± 12.7 ^a^	437.0 ± 3.4^e^	6355.0 ± 191.9 ^g^

Values are mean ± SE (*n* = 3). ^a–g^ Different letter in the same column indicate a significant difference (*p* ≤ 0.05) between the samples. ^A^ Corrected value taking into account the soluble solid loss during cooking. * The asterisk indicates a significant difference between the raw and boiled sample.

**Table 7:** Effect of boiling on physicochemical characteristics of edible plants.

*Scientific Name Plant*	Treatment	WRC g/g	Viscosity (MPa)	Color		
				L *	a *	b *
*Chenopodium nuttalliae* Safford	Raw	11.4 ± 0.8 ^ab^	6.4 ± 0.0 ^d*^	34.9 ± 0.8 ^g*^	−9.1 ± 0.0 ^a*^	22.9 ± 0.1 ^f*^
	Boiled^A^	9.4 ± 1.2 ^ab^	3.8 ± 0.0 ^b^	19.6 ± 0.3 ^b^	−4.0 ± 0.1 ^d^	9.1 ± 0.2 ^c^
*Suaeda torreyana* S. Watson	Raw	12.2 ± 1.1 ^b^	7.7 ± 0.0 ^e*^	28.3 ± 0.6 ^e^	−5.7 ± 0.1 ^c^	9.8 ± 0.2 ^d^
	Boiled^A^	11.3 ± 2.4 ^ab^	3.7 ± 0.2 ^b^	21.9 ± 0.4 ^c^	−4.1 ± 0.1 ^d^	10.1 ± 0.3 ^d^
*Portulaca oleracea* L.	Raw	10.5 ± 0.4 ^ab^	6.5 ± 0.0 ^d*^	33.7 ± 0.2 ^f*^	−3.8 ± 0.1 ^d*^	12.3 ± 0.3^d*^
	Boiled^A^	8.6 ± 1.3 ^a^	5.1 ± 0.0 ^c^	23.8 ± 0.2 ^d^	−1.0 ± 0.0 ^g^	7.8 ± 0.1 ^b^
*Chenopodium album* L.	Raw	12.4 ± 1.3 ^b^	7.7 ± 0.0 ^e*^	24.1 ± 0.2 ^d^	−3.4 ± 0.0 ^e^	10.0 ± 0.1 ^d^
	Boiled^A^	10.1 ± 1.6 ^ab^	5.1 ± 0.0 ^c^	17.6 ± 0.4 ^a^	−2.5 ± 0.1 ^f^	6.8 ± 0.3 ^a^
*Porophyllum ruderale* (Jacq.) Cassini (Asteraceae)	Raw	8.4 ± 0.9 ^a^	2.6 ± 0.0 ^a^	33.5 ± 0.2 ^f^	−8.1 ± 0.0 ^b^	12.8 ± 0.0 ^e^

Values are mean ± SE (*n* = 3). ^a–g^ Different letter in the same column indicate a significant difference (*p* ≤ 0.05) between the samples. ^A^ Corrected value taking into account the soluble solid loss during cooking. * The asterisk indicates a significant difference between the raw and boiled sample.
